# Investigating the effects of medical density on health-seeking behaviours using a multiscale approach to residential and activity spaces: Results from a prospective cohort study in the Paris metropolitan area, France

**DOI:** 10.1186/1476-072X-11-54

**Published:** 2012-12-26

**Authors:** Julie Vallée, Pierre Chauvin

**Affiliations:** 1 UMR Géographie-Cités (CNRS - University Paris 1 - University Paris Diderot), Paris, France; 2 INSERM, UPMC Univ Paris 06, UMRS 707, Paris, France

**Keywords:** France, Daily mobility, Activity space, Polycentric structure, Longitudinal approach, Neighbourhood effects, MAUP, Cancer screening, Health care utilisation

## Abstract

**Background:**

When measuring neighbourhood effects on health, it is both incorrect to treat individuals as if they were static and tied to their residential neighbourhood and to consider neighbourhoods rigid places whose geographical scales can be delineated *a priori*. We propose here to investigate the effects of residential medical density on health-seeking behaviours, taking into account the mono/polycentric structure of individual activity space (i.e., the space within which people move in the course of their daily activities) and exploring various neighbourhood units based on administrative delineations and regular grids.

**Methods:**

We used data collected in the SIRS cohort study, which was carried out over a 5-year period (2005–2010) among a representative population living in 50 census blocks in the Paris metropolitan area. In the 662 women who lived in the same census blocks during the follow-up period and who had reported a recent cervical screening at baseline, we studied the association between residential medical density and individual activity space and the incidence of delayed cervical screening (> 3 years) in multilevel logistic regression models after adjustment for potential confounders.

**Results:**

Among the 662 women studied, there were 94 instances of delayed cervical screening in 2010 (14%). The women who indicated that their activity space was concentrated within their neighbourhood of residence were significantly more at risk for an incident delayed cervical screening. No significant association was found between residential medical density and the incidence of delayed cervical screening. However, we observed a significant interaction between individual activity space and residential medical density. Indeed, women living in neighbourhoods with a low medical density had a significantly higher risk of delayed screening, but only if they reported that their daily activities were centred within their neighbourhood of residence. Lastly, a sensitivity analysis exploring various neighbourhood spatial units revealed that the incidence of delayed screening was better modelled when residential medical densities were calculated from a 1400 × 1400 metre grid or from adjacent census blocks.

**Conclusion:**

This analysis underscores the view that people and neighbourhoods should be considered interacting entities. Using unsuitable neighbourhood units or neglecting the mono/polycentric structure of activity space would result in downplaying the importance of access to local health resources when addressing inequalities in health-seeking behaviours.

## Background

Health-seeking behaviours have often been linked to health services density at the national level [1-4]. At the metropolitan level, it has been noted that the health services density in urban neighbourhoods has significant limitations because this measure does not take individual daily mobility into account [5]. As pointed out recently, the relationship between environmental exposures and individuals and their corresponding health-seeking behaviours has traditionally been situated in the neighbourhood of residence [6,7]. However, it would be incorrect to assume that every urban resident remains static and tied to his/her residential neighbourhood. When studying place-based effects on health, the monocentric people paradigm, structured only around the residential neighbourhood, should not be used. It might be then interesting to integrate people’s activity space (i.e., the space within which they move about or travel in the course of their daily activities) when studying the determinants of health inequalities. In this research, we propose to define the structure of people’s activity space from the respondents’ statements about the location of their usual activities, i.e., within or outside their neighbourhood of residence. From this self-reported measure, we aim to differentiate the respondents whose everyday places were spatially concentrated around a single anchor residential space from those whose everyday places were structured around a polycentric network with several anchor spaces [8].

It has also been pointed out, with regard to measuring residential neighbourhood effects on health, that they can be affected by how neighbourhoods are spatially delineated [9,10]. We therefore decided to calculate the medical density in various neighbourhood units based on administrative delineations and regular grids. These densities were obtained by dividing the number of practitioners by the resident population.

This empirical research was based on data from a cohort study carried out over a 5-year period (2005–2010) in the Paris metropolitan area. Focusing more specifically on cervical cancer screening, we limited our sample to women (i) who lived in the same place during the entire cohort study period and (ii) who, at baseline, had reported having recently undergone cervical screening (< 3 years). Using this prospective design, we propose here to study the effects of residential medical density on the change in cervical screening frequency, taking into account both the structures of individual activity space and various neighbourhood units.

## Materials and methods

### Study population and follow-up

The SIRS (French acronym for health, inequalities and social ruptures) cohort study is a social and epidemiological longitudinal survey carried out in the Paris metropolitan area among a representative sample of the adult French-speaking population. The first two waves of this cohort were conducted in the fall of 2005 and the winter of 2010. A questionnaire containing a large number of social and health-related questions was administered face-to-face during home visits.

The SIRS survey employed a stratified, multistage cluster sampling procedure. The primary sampling units were census blocks called “IRISs” (“IRIS” is a French acronym for blocks for incorporating statistical information). In all, 50 census blocks were selected from the 2595 eligible census blocks in Paris and its suburbs. In 2005, 60 households in each selected census block were randomly chosen from a complete list of households, and one adult was randomly selected from each household by the birthday method. The final sample in 2005 consisted of 3023 people, including 1843 women [11].

Of the 1843 women interviewed in 2005, 839 (45.5%) were living in the same census block in 2010 (even though they may have moved within their block) and were reinterviewed, while 281 (15.2%) were no longer in the same census block as in 2005, 38 (2.1%) had died, 36 (2.0%) were too sick to answer our questions, 42 (2.3%) were away from home during the survey period, 376 (12.4%) declined to answer, and 231 (7.6%) were lost to follow-up (Table 1). We observed that the rate of women who declined to answer and of those lost to follow-up in 2010 was significantly higher among foreigners, women living in low-income households and those with delayed cervical screening in 2005, but that it was similar according to the structure of the women’s activity space.

**Table 1:** **Description of the women followed in the SIRS cohort study** (**2005**–**2010**)

	**Women surveyed in 2005 n=1843, Count (%)**
Interviewed in 2010 and still living in the same census block (residents)	839 (45.5)
Moved between 2005 and 2010 (movers)	281 (15.2)
Died between 2005 and 2010	38 (2.1)
Too sick to answer in 2010	36 (2.0)
Away from place of residence in 2010	42 (2.3)
Declined to answer in 2010	376 (12.4)
Lost to follow-up in 2010	231 (7.6)

Lastly, of the 839 women who were followed from 2005 to 2010 and who were still living in the same census block, we limited the prospective study to those who had indicated, in 2005, that they had undergone cervical screening in the previous three years. The final sample for studying neighbourhood effects on the incidence of delayed cervical screening therefore consisted of 662 women (aged 18 to 84 years in 2005) living in 50 census blocks in the Paris metropolitan area, with a median of 14 women per census block (range: 4 to 25).

### Measures

#### Cervical screening as an example of a preventive health-seeking behaviour

Cervical cancer screening with a Papanicolaou (Pap) smear is the key strategy for the early detection of this type of cancer [12]. In France, gynaecologists perform—in independent, primary care practices—the vast majority of cervical screening tests, even though general practitioners can perform or order such tests, too. Since 1995, in France, the recommendation has been for women to have a Pap test every three years after two normal annual smears [13]. We decided to use a 3-year threshold to divide the adult female population into two subpopulations (three years or less and more than three years since their last Pap test). In the SIRS survey, the date of the last screening test was self-reported by the women.

#### Measurement of activity space

**Figure 1:**
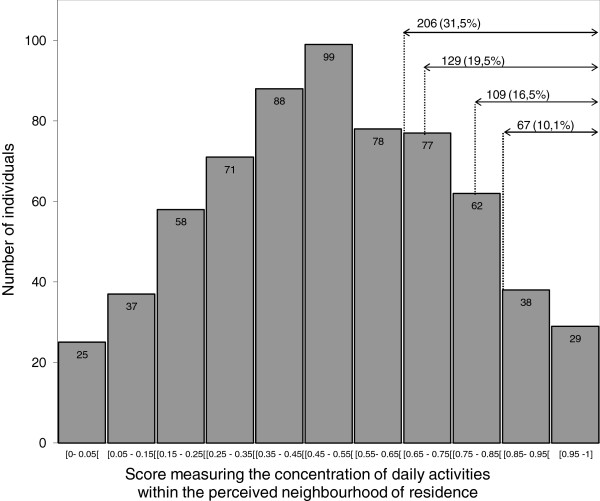
Histogram of the distribution of the score measuring the concentration of daily activities in the perceived neighbourhood.

In this paper, activity space was measured from the respondents’ 2005 statements about the location of some of their domestic and social activities within or outside their neighbourhood of residence [14,15]. The neighbourhood of residence was not defined, and its boundaries were left to the individual’s own assessment and perception. The respondents were asked where they usually 1) go food shopping; 2) use services (bank, post office); 3) go for a walk; 4) meet friends; and 5) go to a restaurant or a café. Activities said to be done “mainly within the neighbourhood of residence” were assigned a value of 1, while those done “both within and outside the neighbourhood” or “mainly outside the neighbourhood” were assigned a value of 0.5 and 0, respectively. By adding these values together and dividing the sum by the total number of reported activities, we obtained an individual score measuring the concentration of daily activities in the perceived neighbourhood of residence (Figure 1). The respondents were then ranked on the basis of this score, which ranged from 0 (for those who reported doing all the activities of interest mainly outside their neighbourhood of residence) to 1 (for those who reported doing all the activities of interest mainly within their neighbourhood of residence). If we use a score threshold value of 0.7, 129 of the 662 women studied (19.5%) can be considered as concentrating their activity space within their neighbourhood of residence, while 553 (83.5%) can be considered as having a polycentrically activity space. Naturally, the proportion of women considered as concentrating their activity space within their neighbourhood of residence changes according to the threshold value chosen (e.g., from 10.1% for a threshold of 0.85 to 31.5% for a threshold of 0.65, as seen in Figure 1).

#### Residential neighbourhood medical density

The precise location of all the practitioners was obtained from the Institute of Development and Urban Planning of the Paris Region (IAURIF) GIS database for Paris and its surrounding departments. This geodatabase was computed from the practitioners’ addresses, which had been exhaustively compiled by Regional Union of Health Insurance Fund (URCAM) in 2009. For this paper, we focused on general practitioners and gynaecologists, both of whom perform cervical screening tests in France.

**Figure 2:**
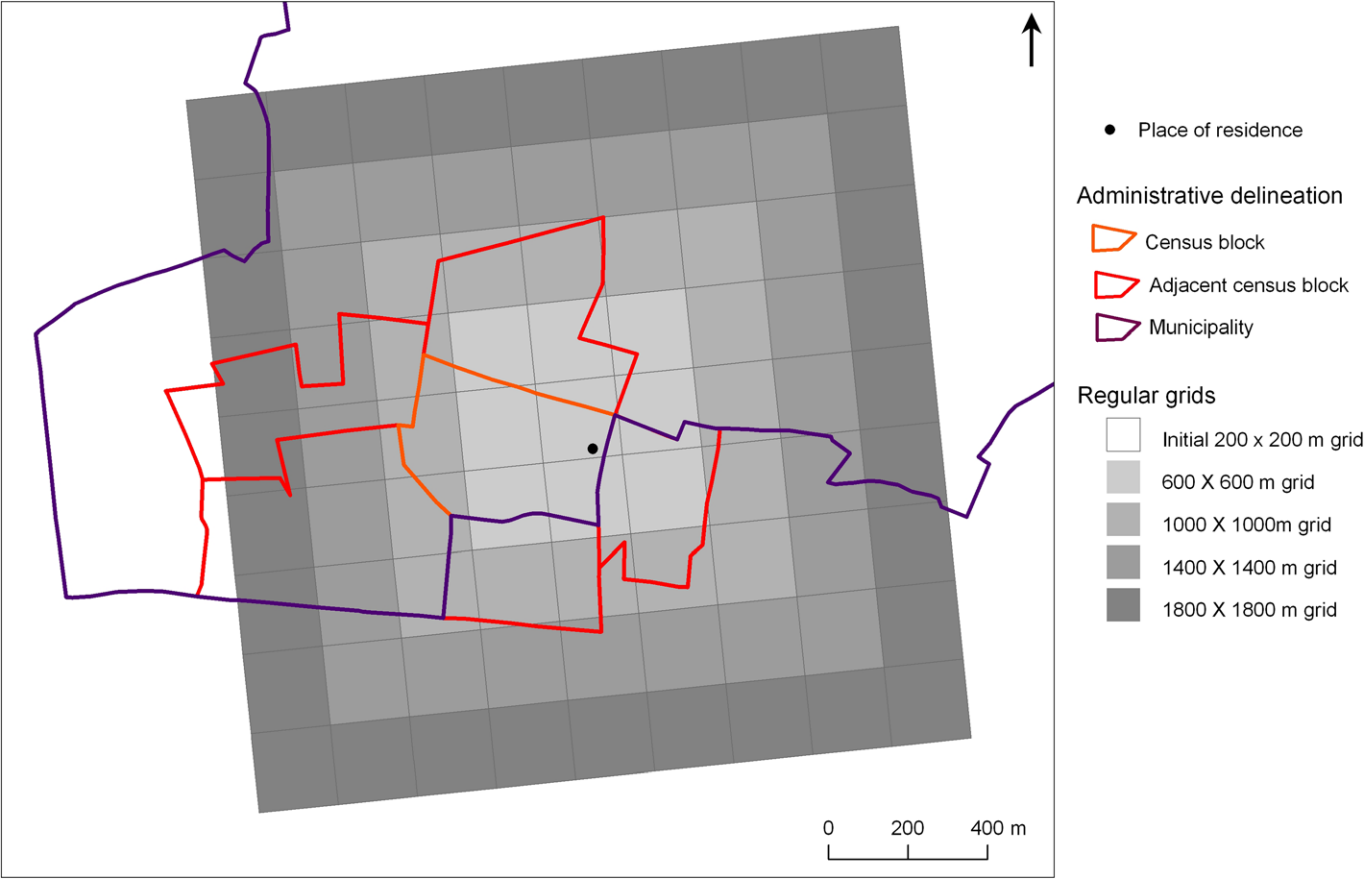
Example showing the spatial superimposition of the seven neighbourhood units for one respondent living in the municipality of Montreuil.

To study neighbourhood medical density, we decided to use various neighbourhood areal units to avoid an *a priori* determination of the geographical zoning and scale. Actually, we considered neighbourhood units on two different bases: (i) administrative delineations, and (ii) regular grids (Figure 2).

– (i) For administrative delineations, we calculated the medical density in three different administrative units: census blocks, groups of adjacent census blocks, and municipalities. The number of general practitioners and gynaecologists included in these administrative areas was divided by the resident population, as reported by the 2009 census. The census blocks, adjacent census blocks and municipalities had a mean area of 0.3, 2.5 and 6.3 km^2^, a mean population of 2489, 16,305 and 71,531, and an average of 134, 93 and 130 general practitioners and gynaecologists per 100,000 population, respectively (Table 2).

**Table 2:** Description of the seven neighbourhood units

			**n**	**Area, in km**^**2**^	**Population**	**Medical density (general practitioners and gynaecologists per 100,000 population)**
				**Mean (Min-Max)**		
Neighborhood units	Based on administrative areas	Census blocks	50	0.31 (0.04-1.80)	2489 (1160–4208)	134.2 (0–3506.2)
		Adjacent census blocks	50	2.55 (0.31-13.54)	16,305 (9294–27,992)	92.8 (18.2-345.8)
		Municipalities	41	6.3 (1.24-16.43)	71,531 (7017–236,491)	102.9 (43.3-265.8)
	Based on regular grids	600 × 600 metres^1^	264	0.36 (0.36-0.36)	4872 (485–18,996)	84.7 (0–895.2)
		1000 × 1000 metres^1^	264	1 (1–1)	12,969 (2156–48,442)	109.7 (0–1976.5)
		1400 × 1400 metres^1^	264	1.96 (1.96-1.96)	22,778 (4155–89,511)	110.3 (17.7-950.7)
		1800 × 1800 metres^1^	264	3.24 (3.24-3.24)	34,071 (3741–132,417)	108.1 (14.7-558.5)

^1^ From an initial 200 × 200 metre grid. Every grid was centred on the cell corresponding to respondent’s place of residence.

– (ii) Using an initial 200 × 200 metre grid indicating the population (provided by the French National Institute of Statistics and Economic Studies - INSEE- according to the 2009 income tax database), we calculated the medical density for four different grid sizes (in metres): 600 × 600; 1000 × 1000; 1400 × 1400 and 1800 × 1800. These grids were systematically centred on the cell corresponding to respondent’s place of residence. These four different grids contained 9, 25, 49 and 81 cells with a side length of 200 metres, for an area of 0.36, 1, 19.96 and 3.24 km^2^, respectively. The mean population in these four grids was 4872, 12,969, 22,778 and 34,071, with an average of 85, 110, 110 and 108 general practitioners and gynaecologists per 100,000 population, respectively (Table 2).

To permit comparisons between the various neighbourhood areal units, the practitioner density was systematically divided into three categories according to the distribution tertiles, and greater attention was paid to the lowest tertile.

#### Other potential individual predictors

We also considered the women’s age, nationality, level of education, employment status and health insurance status and the monthly household income (range: 140 to 8670 € per consumption unit; tertile values: 1270/ 2000 €/CU) as reported in 2005. To calculate the monthly household income, we added up the individual incomes of all the members of the household, or consumption unit (CU), and divided this sum by the adjusted number of people living there.

### Statistical methods

First, we estimated the potential selection bias related to residential mobility by comparing the profile of the women who lived in the same census block between 2005 and 2010 (residents) with that of the women who moved to another census block during that period (movers). Second, we evaluated the association between residential medical density and individual activity space and the incidence of delayed cervical screening in bivariate analyses (when comparing the incidence rates in the subgroups using the chi-square statistic) and multilevel logistic regression models (using the xtmelogit command in Stata11, specifying that the collected data were clustered by census block). In these models, we observed associations between neighbourhood medical density and individual activity space and our outcome after adjustment for potential confounders. Third, we studied the interaction between individual activity space and residential medical density on the incidence of delayed screening and created a variable combining both of them to estimate its association with this outcome after adjustment for potential confounders. Lastly, we systematically performed sensitivity analyses to compare association estimates according to (i) the seven neighbourhood areal units used to calculate the medical densities, and (ii) the four threshold values used to categorize activity space. These models were compared with the Akaike information criterion (AIC). The model with the minimum AIC value was chosen as the one that best fit the data. A *p*-value < 0.05 was used as the significance level for all the statistical analyses presented, except for the interaction term, where a *p*-value < 0.10 was considered significant.

## Results

When comparing the resident and mover subpopulations, we observed that the prevalence of delayed cervical screening (> 3 years) as reported in 2005 was not statistically different, it being, respectively, 20.9% and 18.9% (Table 3). Nor was any significant difference found for activity space or nationality and future residential mobility. However, we noted that young women, women who were working and those with a postsecondary education moved in greater numbers percentage-wise during this 5-year period.

**Table 3:** **Comparison of the women**’**s profiles according to their residential trajectories between 2005 and 2010**

	**Residents: women who were still living in the same census block, n=839**	**Movers: women who moved between 2005 and 2010, n=281**	***Difference (p-value)***
	Count (%)		
**Age**			
18-29 years	83 (9.9)	90 (32.0)	<*0*.*001*
30-44 years	273 (32.5)	109 (38.8)	
45-59 years	260 (31.0)	51 (18.1)	
≥ 60 years	223 (26.6)	31 (11.0)	
**Nationality**			
French	747 (89.0)	243 (86.5)	>*0*.*05*
Foreign	92 (11.0)	38 (13.5)	
**Level of education**			
Postsecondary	405 (48.3)	158 (56.2)	*0*.*05*
Secondary school	346 (41.2)	101 (35.9)	
None or primary school only	88 (10.5)	22 (7.8)	
**Current employment status**			
Working or studying	485 (57.8)	206 (73.3)	<*0*.*001*
Unemployed	60 (7.1)	21 (7.5)	
Housewives	100 (11.9)	26 (9.2)	
Retired	194 (23.1)	28 (10.0)	
**Activity space structure **(*as defined using a 0*.*7 threshold value*)			
Polycentric	661 (79.0)	235 (83.9)	>*0*.*05*
Centred within residential neighbourhood	176 (21.0)	45 (16.1)	
**Date of last cervical screening test**			
Recent (≤ 3 years)	662 (79.1)	228 (81.1)	>*0*.*05*
Delayed (> 3 years)	175 (20.9)	53 (18.9)	

Among the residents who, at baseline, had reported a cervical screening test in the previous three years (n=662), there were 94 reported incident cases of delayed cervical screening in 2010 (14.2%). In bivariate analyses (Table 4), this incidence was significantly higher among women aged 60 or older (27.3%), women of foreign nationality (23.6%), those with no or only a primary school education (37.7%), those living in a household with a low monthly income (20.8%), housewives (23.8%), women who were retired (29.2%), and women who were not fully covered by health insurance in 2005 (25.3%). In one multilevel logistic regression model, we found that three of these factors (age, level of education and employment status) remained statistically associated with the incidence of delayed cervical screening (Table 4, Model 1). Retired women and women aged 60 and over were both found to be at higher risk for delayed screening. Since these two characteristics were closely linked, we decided to keep age only in the subsequent models, in addition to the level of education (Model 2 in Table 4 and Tables 5, 6 and 7).

**Table 4:** **Incidence of delayed cervical screening** (>**3 years**) **between 2005 and 2010 according to individual factors (as reported in 2005) and residential medical density**

	**Sample size**	**% with delayed cervical screening**	**Difference (p-value)**	**Adjusted odds ratio (95% CI)**	
**Total**	662	14.2		*Model 1*	*Model 2*
**Age**					
18-29 years	59	15.2	< 0.001	2.38 (0.92-6.18)	2.86 (1.15-7.14)*
30-44 years	250	12.4		1.83 (0.93-3.63)	1.89 (0.98-3.64)*
45-59 years	225	8.4		Ref.	Ref.
≥ 60 years	128	27.3		2.55 (0.97-6.69)	4.53 (2.31-8.87)**
**Nationality**					
French	590	13.0	< 0.01	Ref.	-
Foreign	72	23.6		1.02 (0.47-2.21)	-
**Level of education**					
Postsecondary	340	8.2	< 0.001	Ref.	Ref.
Secondary school	269	17.1		1.53 (0.82-2.85)	2.17 (1.26-3.74)**
None or primary school only	53	37.7		3.30 (1.37-7.98)**	5.33 (2.44-11.61)**
**Monthly household income**					
High (2001–8670 €/CU)	219	9.1	< 0.01	Ref.	-
Intermediate (1270–2000 €/CU)	222	12.6		1.26 (0.63-2.55)	-
Low (140–1269 €/CU)	221	20.8		1.78 (0.80-3.99)	-
**Current employment status**					
Working or studying	420	8.3	< 0.001	Ref.	-
Unemployed	52	15.4		1.15 (0.46-2.87)	-
Housewives	84	23.8		1.92 (0.93-3.97)	-
Retired	106	29.2		2.84 (1.06-7.64)*	-
**Health insurance coverage**					
Fully covered	587	12.8	< 0.01	Ref.	-
Not fully covered	75	25.3		1.53 (0.73-3.22)	-
**Activity space structure **(*as defined using a 0*.*7 threshold value*)					
Polycentric	533	12.4	< 0.01	Ref.	Ref.
Centred within residential neighbourhood	129	21.7		1.91 (1.08-3.35)*	2.10 (1.20-3.67)**
**Density of general practitioners and gynaecologists in the neighbourhood** (*based on a 1400 x 1400 metres grid*)					
Highest and middle tertiles	437	12.1	0.03	Ref.	Ref.
Lowest tertile	225	18.2		1.35 (0.78-2.33)	1.56 (0.89-2.75)

Results of bivariate analyses and multilevel logistic regression models.

* p < 0.05; **p < 0.01.

**Table 5:** Sensitivity analysis of the association between cervical screening and activity space according to the categorization of individual activity space

		**Structure of activity space**	**Sample size**	**% with delayed cervical screening**	***Difference (p-value)***	**aOR (95% CI)**^***1***^	**AIC**
*Threshold value used to categorize individual activity space*	0.65	Polycentric	456	12.3	< 0.05	Ref.	499.99
		Centred within residential neighbourhood	206	18.4		1.84 (1.09-3.12)*	
	0.7	Polycentric	533	12.4	< 0.01	Ref.	498.73
		Centred within residential neighbourhood	129	21.7		2.10 (1.20-3.67)**	
	0.8	Polycentric	553	13.2	NS	Ref.	502.58
		Centred within residential neighbourhood	109	19.3		1.67 (0.91-3.06)	
	0.85	Polycentric	595	13.6	NS	Ref.	504.49
		Centred within residential neighbourhood	67	19.4		1.38 (0.68-2.82)	

Results of the bivariate analyses and multilevel logistic regression models.

^1 ^Adjusted odds ratio accounting for age, level of education and medical density (based on a 1400 x 1400 metres grid).

**Table 6:** Sensitivity analysis of the association between cervical screening and residential medical density according to the delineation of residential neighbourhoods

			**Medical density tertiles**	**Sample size**	**% with delayed cervical screening**	***Difference (p-value)***	**aOR (95% CI)**^***2***^	**AIC**
*Neighbourhood units*	Administrative areas	Census blocks	Highest and middle	440	15.0	NS	Ref.	501.11
			Lowest	222	12.6		0.91 (0.51-1.61)	
		Adjacent census blocks	Highest and middle	437	13.7	NS	Ref.	501.09
			Lowest	225	15.1		1.11 (0.63-1.96)	
		Municipalities	Highest and middle	424	13.4	NS	Ref.	501.09
			Lowest	238	15.5		1.11 (0.63-1.94)	
	Regular grids	600 × 600 metres^*1*^	Highest and middle	441	13.6	NS	Ref.	500.82
			Lowest	221	15.4		1.19 (0.69-2.04)	
		1000 × 1000 metres^*1*^	Highest and middle	440	13.9	NS	Ref.	501.21
			Lowest	222	14.9		0.97 (0.56-1.67)	
		1400 × 1400 metres^*1*^	Highest and middle	437	12.1	< 0.05	Ref.	498.73
			Lowest	225	18.2		1.56 (0.89-2.75)	
		1800 × 1800 metres^*1*^	Highest and middle	434	13.1	NS	Ref.	501.15
			Lowest	228	16.2		1.08 (0.62-1.86)	

Results of the bivariate analyses and multilevel logistic regression models.

^1 ^From an initial 200 x 200 metres grid. Every grid was centred on the cell corresponding to respondent’s place of residence.

^2 ^Adjusted odds ratio accounting for age, level of education and activity space structure (as defined using a 0.7 threshold value).

**Table 7:** Cross-sensitivity analysis of the interaction between activity space and residential medical density on cervical screening

			***Threshold value used to categorize the respondents’ activity space***											
			**0.65**			**0.7**			**0.8**			**0.85**		
			**Inter-action**^***2***^	**aOR**^***3***^	**AIC**	**Inter-action**^***2***^	**aOR**^***3***^	**AIC**	**Inter-action**^***2***^	**aOR**^***3***^	**AIC**	**Inter-action**^***2***^	**aOR**^***3***^	**AIC**
*Neighbourhood units*	Based on admini-strative areas	Census blocks	p > 0.10	Ref.	504.26	p > 0.10	Ref.	501.43	p > 0.10	Ref.	504.05	p > 0.10	Ref.	505.72
				0.83			1.08			1.05			0.97	
				1.63			2.45*			2.04*			1.76	
				1.67			1.09			0.59			0.35	
		Adjacent census blocks	p > 0.10	Ref.	502.18	**p **= **0**.**09**	Ref.	500.23	p > 0.10	Ref.	505.71	p > 0.10	Ref.	506.26
				0.87			0.88			0.95			0.91	
				1.37			1.48			1.32			0.93	
				2.69*			3.62**			2.42			2.62	
		Municipalities	p > 0.10	Ref.	504.32	p > 0.10	Ref.	503.09	p > 0.10	Ref.	506.23	p > 0.10	Ref.	508.28
				1.12			1.10			1.20			1.10	
				1.72			1.96*			1.86			1.31	
				2.03			2.25*			1.49			1.52	
	Based on regular grids	600 × 600 metres^*1*^	p > 0.10	Ref.	503.36	p > 0.10	Ref.	501.60	p > 0.10	Ref.	505.39	p > 0.10	Ref.	506.34
				1.03			1.01			1.02			0.99	
				1.52			1.64			1.30			0.93	
				2.49*			3.20**			2.57			2.58	
		1000 × 1000 metres^*1*^	p > 0.10	Ref.	504.33	p > 0.10	Ref.	503.08	p > 0.10	Ref.	506.24	p > 0.10	Ref.	508.35
				1.08			0.92			0.86			0.93	
				1.90*			1.86*			1.38			1.33	
				1.58			2.13			1.84			1.26	
		1400 × 1400 metres^*1*^	p > 0.10	Ref.	501.74	**p **= **0**.**08**	Ref.	497.58	**p **= **0**.**09**	Ref.	501.66	p > 0.10	Ref.	505.44
				1.45			1.24			1.25			1.34	
				1.67			1.44			1.13			1.03	
				3.15**			5.05**			4.20**			2.98	
		1800 × 1800 metres^*1*^	p > 0.10	Ref.	504.43	p > 0.10	Ref.	501.39	p > 0.10	Ref.	505.81	p > 0.10	Ref.	507.60
				1.11			0.89			0.94			0.95	
				1.79			1.52			1.29			1.03	
				1.91			2.94*			2.21			1.90	

Results of the multilevel logistic regression models.

^1^ From an initial 200 x 200 metres grid. Every grid was centred on the cell corresponding to respondent’s place of residence.

^2^ Interaction term between medical density and activity space.

^3^ Adjusted odds ratio accounting for age and level of education corresponding to the following four categories: Polycentric activity space and high or medium medical density (used as the reference category); Polycentric activity space and low medical density; Activity space centred within the neighbourhood of residence and high or medium medical density; Activity space centred within the neighbourhood of residence and low medical density.

Women whose activity space was centred within their neighbourhood of residence were significantly more at risk for an incident delayed cervical screening test, as seen in bivariate analysis as well as in multilevel logistic regression models (Table 4). In sensitivity analysis (Table 5), we observed that the associations between activity space and the incidence of delayed cervical screening were statistically significant when the threshold values 0.65 and 0.7 were used to categorize activity space but that they were not significant when the threshold values 0.8 and 0.85 were used. When we compared the AIC values, the best model was found to be the one that included activity space categorized with a threshold value of 0.7.

In the bivariate analyses (Table 6), we observed a significantly higher incidence of delayed cervical screening among women living in a neighbourhood with a low practitioner density (18.2%) than among the others (12.1%), but only if the neighbourhood units were defined as 1400 × 1400 metre grids (Table 6). In multilevel logistic regression models adjusted for age and education level, no significant association was found between medical density and the incidence of delayed cervical screening, regardless of the neighbourhood delineation (Table 6). When we compared the AIC values, the best model was found to be the one with a medical density determined from a 1400 × 1400 metre grid.

Lastly, the interaction between individual activity space and residential medical density on the incidence of delayed screening was found to be statistically significant (with a significance level of 0.10) in multilevel logistic regression models after adjustment for age and level of education, but only when the threshold values 0.7 or 0.8 were used to categorize activity space and when the neighbourhood was defined either from a 1400 × 1400 metre grid or from adjacent census blocks (Table 7). This result underscores the view that individual activity space and residential medical density should not be considered independent factors and that they should be seen as having a combined influence on women’s screening. When we compared the models using the AIC values (Table 7), the best model was found to be the one with activity space categorized with a threshold value of 0.7 and a medical density determined from a 1400 × 1400 metre grid, the second best model being the one with the same activity space categorization but with the medical density determined from the group of adjacent census blocks (Table 7). In both of these models, we observed that the women with a limited activity space who were living in a neighbourhood with a low practitioner density had a significantly higher risk of incident delayed cervical screening (aOR=5.05, 95% CI: 1.95-13.08; and aOR=3.62, 95% CI 1.37-9.56, respectively).

## Discussion

### Crossing individual activity space and residential medical density

We observed in the Paris metropolitan area that the incidence of delayed cervical screening was higher among women who had reported concentrating their activity space within their residential neighbourhood. We also noted that the women who were living in an area with a low medical density had a significantly higher risk of incident delayed cervical screening, but only when their activity space was centred within their residential neighbourhood. For these women, the lack of health resources in their residential neighbourhood really matters, since they have no experience (by choice or by circumstance) moving outside of their residential neighbourhoods in the course of their daily activities, with the result that their health-seeking behaviours are highly influenced by the opportunities and constraints of their residential neighbourhoods. These two findings are consistent with those of a previous analysis based on the cross-sectional data collected in 2005 in the SIRS cohort study [14]. This research in the form of a prospective study may help overcome the causality interpretation problems affecting cross-sectional studies and help confirm the combined effects of individual activity space and residential medical density on participation in preventive health-care activities. In this study, we limited the study sample to women who (i) lived in the same census block over a 5-year period, to reduce biases related to insufficient neighbourhood exposure; and (ii) who had reported an appropriate health-seeking behaviour (i.e., cervical screening no more than three years earlier) at baseline, to observe the determinants of any potential change in cervical cancer screening frequency.

### Exploring various neighbourhood spatial units

For the neighbourhood units based on administrative delineations, our sensitivity analysis revealed that adjacent census blocks were more relevant than census blocks or municipalities for identifying the effects of residential medical density on health. When we compared various neighbourhood units based on administrative delineations and regular grids (with side lengths from 600 to 1800 metres), the neighbourhood medical density determined from 1400 × 1400 metre grids appeared to best model changes in the health-seeking behaviour of interest. Regular grids—and, to a lesser extent, adjacent census blocks—do, in fact, have the advantage of capturing the environment surrounding the respondent’s place of residence, while neighbourhood units, such as census blocks and municipalities, are affected more by edge effects (cf. Figure 2). Adjacent census blocks and the 1400 × 1400 metre grids have a more or less similar mean area and population (a mean area of 2.55 and 1.96 km^2^ and a mean population of 16,305 and 22,778, respectively), even if, by construct, groups of adjacent census blocks and 1400 × 1400 metre grids are characterized by a higher degree of area variability and a higher degree of population size variability, respectively (as shown in Table 2 when comparing the minimum and maximum values). Exploring the effects of residential medical density by means of a sensibility analysis of seven neighbourhood areal units is, therefore, one of the strengths of this paper. However, it would be incorrect to consider model fit the only reliable criterion for determining the most appropriate neighbourhood unit [10] or to extrapolate our results to other metropolitan areas, neighbourhood characteristics or health outcomes [16].

### Measure of mono/polycentric activity space structure

In this paper, we used a measure of activity space to isolate people who reported concentrating their daily activities within their perceived neighbourhood of residence. Since this measure is directly linked to the respondents’ neighbourhood representation, it was not possible here to distinguish the spatial extent of daily mobility or the perceived neighbourhood delineation [14,15]. However, one of the strengths of this measure is that it provides information about the mono/polycentric structure of people’s activity space. Indeed, we were able to differentiate between people whose everyday places were spatially concentrated around only one anchor space (i.e., their residential space) from those whose activity space included a polycentric network of everyday places structured around several anchor spaces. From these measures, we can then effectively account for the actual people ‘spatial polygamy’ [17].

An alternative method for directly investigating the spatial extent of activity space from GPS data and/or interactive mapping was recently reported [8]. Even if these data are very promising, they requires making sensitive choices for transforming the initial activity space polygon (such as the smallest convex polygon containing every activity destination) into a more meaningful activity place network area.

Lastly, using the score measuring the concentration of daily activities in the perceived neighbourhood of residence, we divided the respondents into two groups to isolate those whose activity space was highly centred within their residential neighbourhood. From sensitivity analysis, we showed that our models’ ability to detect significant associations between activity space and delayed cervical screening was better when the 0.7 threshold value was used. However, concordant results were found when lower or higher threshold values were used, which argues in favour of the internal validity of our results.

### Methodological comments

#### Cohort attrition

A main limitation of this cohort study is the rate of women who declined to answer in 2010 (12.4%) and of those who were lost to follow-up (7.6%). These rates were found to be higher among women of foreign nationality, women living in low-income households and those who had reported delayed cervical screening in 2005. In addition, we observed that young women, women who were working and those with a postsecondary education moved in greater numbers percentage-wise during this 5-year period. This sample selection may have introduced some bias whose impact was difficult to control.

#### Selection bias

When studying the effects of residential medical density on health-seeking behaviours, some selection bias may also occur if individuals with inappropriate health-seeking behaviours are particularly inclined to move into or remain within neighbourhoods with a low medical density and if people with appropriate health-seeking behaviours are particularly inclined to move into or remain in neighbourhoods with a high medical density. This potential selection bias can be considered limited here because no significant association was found between the date of the last cervical screening as reported at baseline and the women’ residential mobility during the next five years.

#### Single time point for analysing residential medical density

The use of a single measure of medical density assumes that no change in medical density had occurred during the 5-year follow-up period, which may be one limitation of this research [18], even if the time period under consideration is relatively short.

#### Self-reported cervical screening

In the SIRS cohort study, the date of the last cervical screening test was self-reported by the women. The accuracy with which women report cervical screening histories may be affected by memory bias and social desirability bias. For cervical screening tests, we can assume that these biases might be less problematic than for other, more-sensitive health-seeking behaviours. Moreover, the entire structure of the long and detailed SIRS social and health questionnaire was designed to reduce any risk of social desirability bias as much as possible.

### Political implications

With regard to urban planning policy, this research underscores the importance of enabling people to overcome any material or physical difficulties so that they can move about outside their neighbourhood of residence. Special efforts in public transportation for populations living in low- medical-density areas could help improve their participation in preventive health-care activities.

As for public health policies, this research suggests that specific measures should be taken in neighbourhoods where the practitioner density is low and where the residents’ activity space is limited and spatially concentrated. We also recommend paying close attention to the geographical scale used to target places underserved by health facilities, since sensitivity scale analysis showed that neighbourhood units that are too small or too large may not be relevant for analysing local access to health facilities. In fact, using unsuitable neighbourhood units or neglecting the structure of individual activity space would result in downplaying the importance of access to local health resources when addressing inequalities in health-seeking behaviours.

Lastly, it would be inaccurate to extrapolate our results to smaller urban settings or to rural areas because our survey population was from France’s largest metropolitan area, which has a particular spatial distribution of health providers [19], patterns of residential trajectories [20] and commuter flows [21]. On the other hand, it would be interesting to replicate the same kinds of prospective analysis in other major urban settings, particularly in other world cities that share similar urban and social patterns with Paris.

## Conclusion

Quantitative studies of place-based effects on health are often implicitly based on an arbitrary distinction between individual characteristics and place characteristics and do not take their interactions into consideration. Yet, people and neighbourhoods should be viewed as dynamic, interacting entities. Our findings, from a prospective cohort study, call for reconsidering the notion of geographical accessibility to health facilities in urban settings on the basis of a multiscale approach to residential and activity spaces.

## Competing interests

The authors declare that they have no competing interest.

## Authors’ contributions

JV analysed the data and wrote this paper. PC contributed to the interpretation of the data and to the writing of the paper. Both authors read and approved the final manuscript.
